# Screw Worms in the Mouth

**Published:** 1890-04

**Authors:** E. Cross

**Affiliations:** San Antonio, Texas


### Correspondence.

### SCREW-WORMS IN THE MOUTH.

San Antonio, Texas, March, 1890.

*Editor Daniel's Texas Medical Journal:*

I saw to-day what to me was a remarkable case. A railroad hand who had been some weeks in the Santa Rosa hospital, suffering from an attack of enteric fever, was convalescent, though a portion of the time lying in semi-unconcious condition. Three or four days ago complained of tooth-ache, for which some simple remedy was prescribed, and I heard nothing more of the trouble until this morning I noticed that the cheek was swollen; examined the gum, supposing there was a collection of pus. When to my surprise, I found quite a cavity and a deep rugged ulcer of the gum over the second and third upper molars, and this filled with screw worms. (What is the technical name?) As many as ten were taken out. They seemed to have burrowed between the roots of the teeth and mucous membrane of the gum. No pain was complained of, and the poor fellow was much disgusted and surprised at finding such animals in his mouth. The cavity was opened; thoroughly washed out, then mopped with pure carbolic acid and filled with iodoform. The nurses told me he slept with his mouth open, and as in such fevers, it was very dry. I suppose the fly deposited its larva upon the gum, and thus followed results as recorded. Did you ever hear of such a case, and is it not somewhat singular that no pain or inconvenience was complained of except one day a slight tooth ache? Yet the worms were quite large and numerous. E. CROSS, M. D.

[Similar cases have been published in this JOURNAL; two, by Dr. F. A. Schmitt, we believe; in his cases the worms were in the nasal cavity, and were expelled by blowing calomel upon them.—ED.]

